# Direct Electron Transfer from Upconversion Graphene Quantum Dots to TiO_2_ Enabling Infrared Light-Driven Overall Water Splitting

**DOI:** 10.34133/2022/9781453

**Published:** 2022-04-13

**Authors:** Dongmei Jia, Xiaoyu Li, Qianqian Chi, Jingxiang Low, Ping Deng, Wenbo Wu, Yikang Wang, Kaili Zhu, Wenhao Li, Mengqiu Xu, Xudong Xu, Gan Jia, Wei Ye, Peng Gao, Yujie Xiong

**Affiliations:** ^1^College of Material, Chemistry and Chemical Engineering, Hangzhou Normal University, Hangzhou, Zhejiang 311121, China; ^2^School of Chemistry and Materials Science, University of Science and Technology of China, Hefei, Anhui 230026, China

## Abstract

Utilization of infrared light in photocatalytic water splitting is highly important yet challenging given its large proportion in sunlight. Although upconversion material may photogenerate electrons with sufficient energy, the electron transfer between upconversion material and semiconductor is inefficient limiting overall photocatalytic performance. In this work, a TiO_2_/graphene quantum dot (GQD) hybrid system has been designed with intimate interface, which enables highly efficient transfer of photogenerated electrons from GQDs to TiO_2_. The designed hybrid material with high photogenerated electron density displays photocatalytic activity under infrared light (20 mW cm^−2^) for overall water splitting (H_2_: 60.4 *μ*mol g_cat._^−1^ h^−1^ and O_2_: 30.0 *μ*mol g_cat._^−1^ h^−1^). With infrared light well harnessed, the system offers a solar-to-hydrogen (STH) efficiency of 0.80% in full solar spectrum. This work provides new insight into harnessing charge transfer between upconversion materials and semiconductor photocatalysts and opens a new avenue for designing photocatalysts toward working under infrared light.

## 1. Introduction

The sun provides 173,000 TW energy every year, 9,000 times more than the annual world energy consumption. While the energy proportion of infrared (IR) light exceeds 50% in sunlight, the IR light has not been efficiently utilized for solar-to-chemical energy conversion by now due to the lack of related ideal materials to photogenerate electrons with sufficient energy and density. As a typical example, solar-driven water splitting, which can produce hydrogen with the energy density of 141.9 MJ/kg, possesses a 0.5 eV overpotential and requires the energy input of at least 1.8 eV corresponding to the photons at <688 nm. To implement IR or near-IR (NIR) (780~2526 nm) light in water splitting, two main strategies have been developed including localized surface plasmon resonance- (LSPR-) induced hot electron injection and photon upconversion-induced electron injection. For instance, a CdS/Cu_7_S_4_ photocatalyst was employed for water splitting, in which LSPR-induced hot electrons from Cu_7_S_4_ were injected into CdS under NIR illumination (>800 nm) [1–3]. Similarly, other photocatalysts were demonstrated based on Au nanostructures with LSPR band in IR spectral region [4–6]. In parallel, photon upconversion is another strategy for utilizing NIR or IR light in solar energy storage and conversion. For instance, core-shell Pt@MOF/Au composites can convert NIR light to UV and visible light, driving photocatalytic hydrogen production under NIR irradiation [7]. Such an (N)IR-driven photocatalysis was also achieved by rare-earth upconversion materials [8–12]. Nevertheless, the energy conversion performance of the existing systems using NIR or IR light is unsatisfactory through the two strategies. The performance of two strategies relies on plasmonic hot electron generation or photon upconversion and, more importantly, their charge or energy transfer to semiconductor photocatalyst. As a matter of fact, the efficiency of these fundamental processes is relatively low, constituting the obstacle for overall performance.

Recently, graphene quantum dots (GQDs), as a class of zero-dimensional nanomaterials based on graphene, have attracted extensive attention owing to their advantages including low toxicity, high electron mobility (10,000 cm^−2^ s^−1^), high carrier concentration (1,013 cm^−2^), low cytotoxicity, and facile surface grafting [13–16]. In addition, the quantum confinement and edge effect of GQDs endow them with more active sites [17, 18], wide optical absorption range [19], tunable band structure, and (N)IR upconversion photoluminescence (PL) behavior [20]. In particular, a prevenient work has demonstrated that the energy difference between excitation light and emission light in the upconversion process is close to 1.1 eV because the p electrons are excited to a high-energy state (e.g., lowest unoccupied molecular orbital, LUMO) and then transition back to the s orbital [21]. We envision that such a upconversion material should be an ideal candidate for forming intimate interface with semiconductor given its facile surface chemistry, which would allow the direct transfer of multiphoton-generated electrons from upconversion GQDs to semiconductor photocatalyst (instead of emitting higher-energy photons) toward (N)IR-driven chemical reactions. Certainly, this opportunity based on upconversion GQDs remains largely unexplored in photocatalysis.

In this work, we demonstrate the concept that the electrons NIR-generated in GQDs through a multiphoton process can be directly transferred to semiconductor toward photocatalytic overall water splitting. As a model system, the surface of 2~3 layered GQDs is modified by reduction treatment and forms intimate bonding with TiO_2_ nanotube photocatalyst, enabling efficient interfacial electron transfer. As a result, the GQDs can offer energy-sufficient electrons under IR irradiation for the photocatalyst to drive water splitting. While the composite displays a strong light absorption form UV region to IR region, the IR activity enhances the photocatalytic performance of overall water splitting to the solar-to-hydrogen (STH) efficiency of 0.80%.

## 2. Results

**Figure 1:**
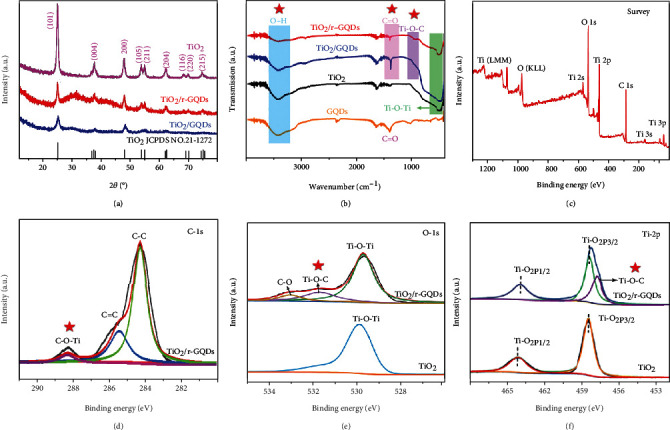
(a) XRD patterns and (b) FT-IR spectra of the obtained GQDs, TiO_2_, TiO_2_/GQDs, and TiO_2_/r-GQDs. XPS spectra of the TiO_2_ and TiO_2_/r-GQDs: (c) survey, (d) C1s, (e) O1s, and (f) Ti2p.

The GQDs are prepared through evolution from glucose and are further treated to remove surface oxygen-containing groups through reduction with NaBH_4_, producing r-GQDs. To demonstrate the importance of interface to charge transfer, the GQDs and r-GQDs with different surface conditions are both used for integration with TiO_2_ nanotubes through the same hydrothermal process.  Figure 1(a) shows the XRD patterns of pure TiO_2_, TiO_2_/GQDs, and TiO_2_/r-GQDs composites. After integrated with TiO_2_, the intensity of the peaks corresponding to TiO_2_ is evidently reduced. This suggests that the crystallinity of TiO_2_ is lowered by the addition of GQDs (r-GQDs) or/and the TiO_2_ is largely covered by the GQDs (r-GQDs). In addition, the average grain size of TiO_2_is calculated to be about 12 nm using Scherrer formula according to its XRD pattern. To gain surface information, Fourier transform infrared spectroscopy (FT-IR) is employed to characterize the samples. As shown in Figure 1(b), the oxygen groups such as OH (~3400 cm^−1^) and C=O (~1400 cm^−1^) in GQDs are obviously reduced after NaBH_4_ reduction. In the meantime, it creates abundant dangling bonds of carbon, which then are bonded with oxygen atoms in TiO_2_ [23, 24]. As a result, the Ti-O-C chemical bonds at 950 cm^−1^ are observed in TiO_2_/r-GQDs [25–27].

The formation of such Ti-O-C chemical bonds is also confirmed by X-ray photoelectron spectroscopy (XPS). As shown in Figure 1(c), the XPS survey spectrum of TiO_2_/r-GQDs composite demonstrates the existence of O, Ti, and C elements. The C1s peaks at 284.6 eV, 285.6 eV, and 288.7 eV are attributed to C-C, C=C and C-O-Ti bonds, respectively (Figure 1(d)) [28]. The Ti-O-C bond is also detected by the peaks at 531.6 eV of O1s (Figure 1(e)) and 457.8 eV of Ti2p (Figure 1(f)) XPS spectra [21]. In comparison, the peaks for Ti-O-C bond have not been found for TiO_2_ and TiO_2_/GQDs (fig. S1). As such, the peak intensity change of TiO_2_ in XRD should also be partially associated with the formation of Ti-O-C chemical bonding between TiO_2_ and r-GQDs. This chemical bonding at the interface of TiO_2_/r-GQDs surely will facilitate the interfacial electron transfer by lowering the potential barrier between TiO_2_ and r-GQDs [29, 30]. It is known that most composites are formed without intimate connection between two different components to leave gaps or defects at the interface, which forms a depletion layer to hinder the charge transfer to a large extent. A chemically bonded interface in the composite undoubtedly provides a better charge transfer path and enables efficient charge transfer from interface to surface.

**Figure 2:**
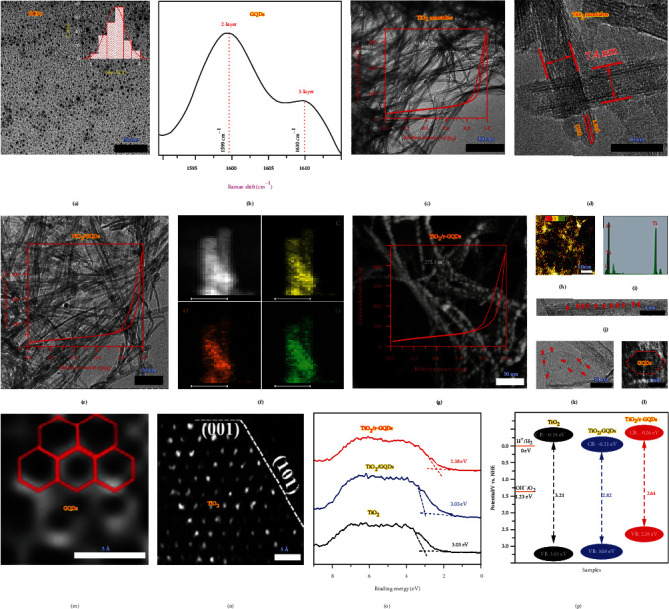
Morphology and microstructure characterization of the samples. (a) TEM image and (b) Raman spectrum of the obtained GQDs. The inset of (a) shows the size distribution. (c) TEM image and (d) HRTEM images of the obtained TiO_2_ nanotubes. The inset of (c) shows the BET result. (e) TEM image and (f) elemental mapping profiles of the obtained TiO_2_/GQDs (scale bars are 10 nm). The inset of (e) shows the BET result. (g) TEM image, (h) elemental mapping profile, (i) EDX spectrum, (j–l) HRTEM images, and (m, n) AC-TEM images of the obtained TiO_2_/r-GQDs. (o) Valence-band spectra measured by XPS and (p) band structures of the three samples.

To reveal the morphologies and microstructures, the samples are examined by transmission electron microscopy (TEM).  Figure 2(a) shows that the as-prepared GQDs have the size of about 2~3 nm. As indicated by Raman spectroscopy (Figure 2(b) and fig. S2), both GQDs and r-GQDs are 2~3 layered thick. Meanwhile, the TiO_2_ nanotubes that are used for integration with GQDs and r-GQDs have a width of ~7.4 nm and a length of several micrometers (Figure 2(c)). High-resolution TEM (HRTEM, Figure 2(d)) reveals that the TiO_2_ nanotubes possess relatively smooth surface. Raman spectroscopy also confirms that the TiO_2_ nanotubes are of anatase phase (fig. S3). After uniformly integrated with GQDs or r-GQDs, the one-dimensional morphology is well maintained as indicated by TEM and elemental mapping (Figures 2(e)–2(i)). Despite the remained nanotubes, the samples display interesting characteristics in Brunauer-Emmett-Teller (BET) surface areas (the insets of Figures 2(c), 2(e), and 2(g)). The TiO_2_ nanotubes and TiO_2_/GQDs exhibit similar BET surface areas (93.8 m^2^/g and 97.2 m^2^/g) and pore size distributions (fig. S4). However, the BET area of TiO_2_/r-GQDs reaches up to 275.3 m^2^/g (Figure 2(g)). Most likely, this feature is associated with the Ti-O-C chemical bonding between TiO_2_ and r-GQDs, which induces the electronegativity change in both TiO_2_ and r-GQDs promoting N_2_ polarization and adsorption. The integrated composite structure of TiO_2_/r-GQDs has also been resolved by HRTEM (Figures 2(j)–2(l)), showing that hexangular r-GQDs are loaded on the surface of TiO_2_ nanotubes. Aberration-corrected TEM (AC-TEM, Figures 2(m) and 2(n)) further confirms the existence of r-GQDs and TiO_2_ at atomic resolution.

The addition of GQDs and r-GQDs broadens the light absorption of TiO_2_ nanotubes to visible region as shown in fig. S5. To determine band structures, valence-band spectra are measured by XPS (Figure 2(o)). As depicted in Figure 2(p), GQDs lower the conduction band (CB) edge from -0.18 eV to +0.21 eV, which is unfavorable for hydrogen evolution through water splitting. However, r-GQDs can elevate the CB and valence band (VB) edges to -0.26 eV and +2.38 eV, respectively, which enable overall water splitting for hydrogen and oxygen production simultaneously. This highlights that the surface modification on GQDs has a significant impact on band structures, which may in turn alter charge dynamics.

**Figure 3:**
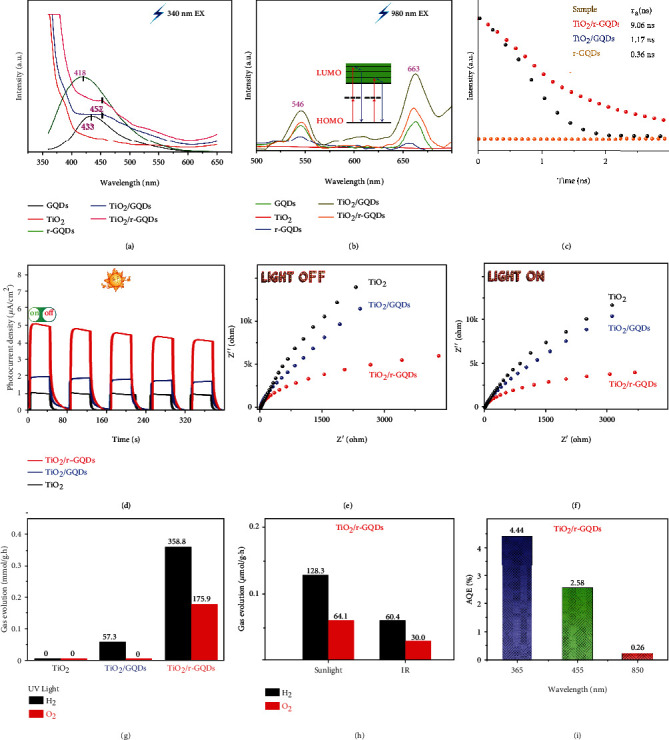
Charge dynamics behavior and photocatalytic performance. PL spectra of the samples with (a) 340 nm and (b) 980 nm excitation. (c) Fluorescence lifetime spectra, (d) transient photocurrent spectra, and (e, f) electrochemical impedance spectra of the TiO_2_, TiO_2_/GQDs, and TiO_2_/r-GQDs. (g, h) Photocatalytic water splitting performance (UV light: 100 mW cm^−2^; sunlight: 100 mW cm^−2^; and IR light: 20 mW cm^−2^). (i) AQE values of TiO_2_/r-GQDs under different illumination wavelengths.

To look into charge dynamics, we closely examine the PL behavior of samples. It is known that the capture centers for excitons are formed by surface oxidation, leading to surface-state-related fluorescence [31]. As shown in Figure 3(a), after reduction of GQDs, the PL emission of graphene quantum dots exhibits an obvious blue-shift from 433 to 418. This observation is similar to the finding in literature that the bandgap is widened as a decreasing number of oxygen atoms are present in the structure, resulting in a PL blue-shift [32]. More importantly, multiphoton upconversion-induced emissions at 546 nm and 663 nm are detected for the GQD-based samples under a 980 nm IR light excitation as shown in Figure 3(b). This upconverted PL property of GQDs should be attributed to the multiphoton active process similar to the previously reported carbon dots [33], indicating that GQDs should be a powerful energy-transfer component in photocatalyst design. The unchanged emission positions between GQDs and r-GQDs demonstrate that the emission should originate from carbon core rather than surface state. After GQDs are anchored on TiO_2_, the sample displays the strengthened upconverted PL emission, suggesting that more active electrons are formed on surface. The time-resolved PL spectra (Figure 3(c)) show that the average PL lifetimes of r-GQDs, TiO_2_/GQDs, and TiO_2_/r-GQDs are 0.3 ns, 1.17 ns, and 9.06 ns, respectively, proving that the photogenerated electrons of r-GQDs can be timely extracted by coupling with TiO_2_.

This argument is also supported by photocurrent measurements as displayed in Figure 3(d). The photocurrents by TiO_2_/r-GQDs are dramatically higher than those by TiO_2_/GQDs and TiO_2_, demonstrating that charge separation and transfer are better harnessed in TiO_2_/r-GQDs. As a supplementary experiment, electrochemical impedance spectroscopy (EIS) measurements (Figures 3(e) and 3(f)) are carried out at a 4 kHz frequency in dark and under 420 nm illumination. It shows that the arc radius under light irradiation is smaller than that in dark. As compared with TiO_2_ and TiO_2_/GQDs, the Nyquist plot of TiO_2_/r-GQDs displays a substantially smaller radius under irradiation, which further proves the improved separation efficiency of electron-hole pairs in TiO_2_/r-GQDs [34]. The enhanced charge separation in TiO_2_/r-GQDs is also demonstrated by surface photovoltage spectroscopy (SPS) (fig. S6). It is known that distinct SPS signal can reflect the enhanced separation rate of photo-induced charge pairs [35]. Among these features, the high conductivity and strong electron transfer ability of r-GQDs facilitate the access to electrons and the electron diffusion process, effectively improving the charge transfer in the photocatalyst. Taken together, the r-GQDs possess the upconversion properties for harvesting IR photons and the electronic properties for efficient charge transfer, which should be a good candidate to offer high photocatalytic activity.

We are now in a position to evaluate the photocatalytic overall water splitting performance of the samples. The measurements are first carried out under ultraviolet light without the addition of precious metal cocatalyst and sacrificial agent. As shown in Figure 3(g), pure TiO_2_ and TiO_2_/GQDs do not have the ability for overall water splitting due to their mismatched energy level structure or unsuitable bandgap (Figure 2(p)). In contrast, TiO_2_/r-GQDs show excellent photocatalytic performance for overall water splitting under UV light with the H_2_ production rate of 358.8 *μ*mol g_cat._^−1^ h^−1^ and the O_2_ production rate of 175.9 *μ*mol g_cat._^−1^ h^−1^. More importantly, TiO_2_/r-GQDs also exhibit photocatalytic activity for overall water splitting under IR light. As shown in Figure 3(h), the photocatalyst offers the values of H_2_ (60.4 *μ*mol g_cat._^−1^ h^−1^) and O_2_ (30.0 *μ*mol g_cat._^−1^ h^−1^) production under IR light (>800 nm, 20 mW cm^−2^). As such, the H_2_/O_2_ production rates of 128.3/64.1 *μ*mol g_cat._^−1^ h^−1^ are achieved in full spectrum (100 mW cm^−2^). To better assess the overall performance, the STH efficiency is determined to be 0.80%. In addition, the apparent quantum efficiency (AQE) of TiO_2_/r-GQDs under different illustration wavelengths (365 nm: 61 mW cm^−2^, 455 nm: 45 mW cm^−2^, and 850 nm: 15 mW cm^−2^) is measured, respectively, as shown in Figure 3(i). The sample displays a 0.26% AQE even under 850 nm infrared light, which further confirms its outstanding ability for overall water splitting. The TiO_2_/r-GQD photocatalyst also shows high stability in cycling tests as shown in fig. S7-S9.

**Figure 4:**
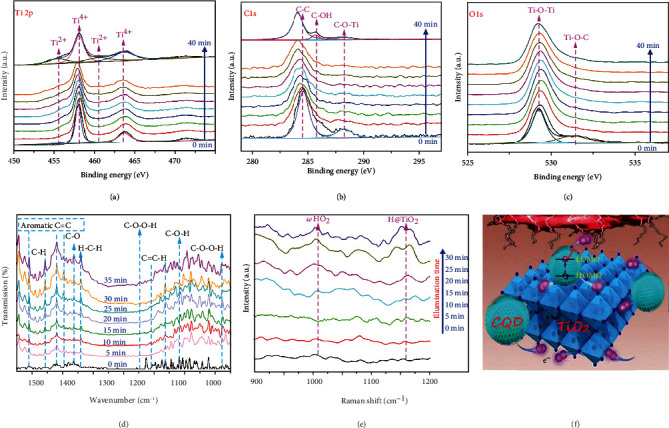
Electron transfer from r-GQDs to TiO_2_ investigated by (a–c) in situ XPS, (d) in situ FT-IR, and (e) in situ Raman measurements. (f) Illustration of the electron transfer process in TiO_2_/r-GQDs.

Upon recognizing the performance, a question naturally arises how the upconversion r-GQDs participate in water splitting after harvesting IR light. To decode the mechanism, we employ in situ XPS to characterize the sample with trace water. As shown in Figure 4(a), the peaks located at 458.0 and 460.6, 455.3, and 463.7 eV are attributed to the Ti^4+^ and Ti^2+^ oxidation states, respectively. The peaks belonging to Ti^2+^ show an increasing trend with the extension of irradiation time. At the same time, the common Ti^3+^ peaks, such as that around 457.0 eV for Ti2p_1/2_, are not distinct [36, 37]. In comparison, only Ti^3+^ is observed for TiO_2_-based photocatalysts under light irradiation [38, 39]. This indicates that the electrons generated from the upconversion of r-GQDs can be directly transferred to TiO_2_. In addition, a shift of C1s peaks toward lower binding energies is observed in Figure 4(b), which should be due to the lowered electron density in carbon atoms after the electron transfer process and further demonstrates the electron transfer from r-GQDs to TiO_2_ under illumination. In the meantime, the O1s peaks assigned to TiO_2_ and Ti-O-C (Figure 4(c)) are unchanged. The water splitting process is also characterized by in situ FT-IR spectroscopy. As shown in Figure 4(d), once the light is turned on, the peaks corresponding to C-H (1458 cm^−1^), C-O stretching in carboxyl (1364 cm^−1^), H-C-H (1340 cm^−1^), and C-O-O-H (1148 cm^−1^ and 878 cm^−1^) are gradually strengthened [40, 41]. The appearance of these transient oxygen-containing species confirms the fact that the oxygen evolution reaction (OER) takes place on CQDs. Simultaneously, the crucial C-O-O-H intermediate detected in water splitting points out a single-site process of OER [36], which is known as the rate-limiting step. In this single-site process, firstly a -OH is bonded with carbon atom of CQD and then loses its hydrogen atom to form a C-O species. Further another -OH is added on C-O for -COOH formation, which finally releases an O_2_ molecule. In addition, the peaks attributed to Ti-H are also found in the measurement (Figure 4(e) and fig. S10), suggesting that hydrogen is produced at the Ti sites in TiO_2_ [42, 43]. The efficient transfer of upconversion-generated electrons from r-GQDs to the Ti sites in TiO_2_ is responsible for the IR-driven overall water splitting as illustrated in Figure 4(f).

It should be noted that such efficient transfer of upconversion electrons can also trigger photocatalytic CO_2_ reduction with IR light. As shown in fig. S11-15, the TiO_2_/r-GQD sample shows 19.49 *μ*mol g_cat._^−1^ h^−1^ CO and 3.13 *μ*mol g_cat._^−1^ h^−1^ CH_4_ production rates under full-spectrum light (100 mW cm^−2^) as well as 0.45 *μ*mol g_cat._^−1^ h^−1^ CO and 0.03 *μ*mol g_cat._^−1^ h^−1^ CH_4_ production rates under weak IR light (>800 nm, 20 mW cm^−2^).

## 3. Discussion

In summary, reduced graphene quantum dots are integrated with TiO_2_ photocatalyst by forming intimate interface, allowing the direct transfer of multiphoton-generated electrons from r-GQDs to TiO_2_ toward IR-driven photocatalysis. The high electron density induced by such a direct electron transfer invests the sample with prominent photocatalytic abilities under infrared light for not only overall water splitting but also CO_2_ reduction. Remarkably, the designed hybrid material achieves photocatalytic overall water splitting for H_2_ at 60.4 *μ*mol g_cat._^−1^ h^−1^ and O_2_ at 30.0 *μ*mol g_cat._^−1^ h^−1^ under infrared light (>800 nm, 20 mW cm^−2^). Such an IR activity makes an important contribution to the STH of 0.80%. This work provides new insights into photocatalyst design for harnessing low-energy photons.

## 4. Materials and Methods

### 4.1. Preparation of TiO_2_ Nanotubes

All the chemicals were of analytical grade. TiO_2_ nanotubes were synthesized using an alkaline hydrothermal process according to the literature [22].

### 4.2. Preparation of GQDs and r-GQDs

Glucose was dispersed in 40 mL pure water. The solution was stirred in a magnetic stirrer for 10 min, then transferred to a Teflon lined autoclave (50 mL), and heated at 190°C for 3 h. After the reaction, the autoclave was naturally cooled to room temperature. The brown solution was centrifuged for 20 min to remove the precipitate and retain the supernatant, namely, GQDs. The aqueous suspension of GQDs (0.1-1 mg/mL) was added with 50 mg NaBH_4_, and the reaction was under stirring at room temperature for 4 h. The resulted product was named as r-GQDs.

### 4.3. Preparation of TiO_2_/GQDs and TiO_2_/r-GQDs

The TiO_2_/GQDs or TiO_2_/r-GQD composites were obtained by the hydrothermal method. 0.2 g TiO_2_ and 40 mL GQDs or r-GQD suspension were mixed. The mixture was continuously stirred at room temperature for 4 h to obtain a uniform suspension. TiO_2_/GQDs or TiO_2_/r-GQD was collected by centrifugation, washed three times with distilled water, and dried in vacuum overnight at 60°C.

## Data Availability

All data needed to evaluate the conclusions in the paper are present in the paper and the Supplementary Materials. Additional data related to this paper may be requested from the authors.

## Supplementary Materials

Supplementary Materials Figure S1: XPS spectra of TiO_2_/GQDs. Figure S2: Raman spectrum of r-GQDs. Figure S3: Raman spectrum of TiO_2_. Figure S4: pore size distributions of TiO_2_/GQDs and TiO_2_/r-GQDs. Figure S5: UV–vis reflectance spectra and optical bandgaps. Figure S6: SPV spectra. Figure S7: time-dependent IR light-driven photocatalytic water splitting by TiO_2_/r-GQDs. Figure S8: cycling IR light-driven photocatalytic water splitting by TiO_2_/r-GQDs. Figure S9: TEM image of TiO_2_/r-GQDs after photocatalytic water splitting. Figure S10: in situ FT-IR spectra. Figure S11: time-dependent simulated sunlight-driven photocatalytic CO_2_ reduction by TiO_2_/r-GQDs. Figure S12: cycling simulated sunlight-driven photocatalytic CO_2_ reduction by TiO_2_/r-GQDs. Figure S13: gas chromatography for CO and CH4 generation. Figure S14: time-dependent IR light-driven photocatalytic CO_2_ reduction by TiO_2_/r-GQDs. Figure S15: cycling IR light-driven photocatalytic CO_2_ reduction by TiO_2_/r-GQDs.
