# Supplementary material for: Direct Electron Transfer from Upconversion Graphene Quantum Dots to TiO2 Enabling Infrared Light-Driven Overall Water Splitting
Source: Research (Wash D C). 2022 Apr 13;2022:9781453. doi: 10.34133/2022/9781453 (PMC9029198; doi:10.34133/2022/9781453)
Supplement: Supplementary Materials — Figure S1: XPS spectra of TiO2/GQDs. Figure S2: Raman spectrum of r-GQDs. Figure S3: Raman spectrum of TiO2. Figure S4: pore size distributions of TiO2/GQDs and TiO2/r-GQDs. Figure S5: UV–vis reflectance spectra and optical bandgaps. Figure S6: SPV spectra. Figure S7: time-dependent IR light-driven photocatalytic water splitting by TiO2/r-GQDs. Figure S8: cycling IR light-driven photocatalytic water splitting by TiO2/r-GQDs. Figure S9: TEM image of TiO2/r-GQDs after photocatalytic water splitting. Figure S10: in situ FT-IR spectra. Figure S11: time-dependent simulated sunlight-driven photocatalytic CO2 reduction by TiO2/r-GQDs. Figure S12: cycling simulated sunlight-driven photocatalytic CO2 reduction by TiO2/r-GQDs. Figure S13: gas chromatography for CO and CH4 generation. Figure S14: time-dependent IR light-driven photocatalytic CO2 reduction by TiO2/r-GQDs. Figure S15: cycling IR light-driven photocatalytic CO2 reduction by TiO2/r-GQDs. [file 9781453.f1.docx]

Supplementary Materials

Title

Direct Electron Transfer from Upconversion Graphene Quantum Dots to TiO_2_ Enabling Infrared Light-Driven Overall Water Splitting

**Authors**

Dongmei Jia^1†^, Xiaoyu Li^1†^, Qianqian Chi^1†^, Jingxiang Low^2^, Ping Deng^1^, Wenbo Wu^1^, Yikang Wang^1^, Kaili Zhu^1^, Wenhao Li^1^, Mengqiu Xu^1^, Xudong Xu^1^, Gan Jia^1^, Wei Ye^1^*, Peng Gao^1*^, and Yujie Xiong^2^*

**Affiliations**

^1^ College of Material, Chemistry and Chemical Engineering, Hangzhou Normal University, Hangzhou, Zhejiang 311121, P. R. China

^2^ School of Chemistry and Materials Science, University of Science and Technology of China, Hefei, Anhui 230026, P. R. China

*Corresponding author. Wei Ye; Email: yewei@hznu.edu.cn; Peng Gao; [gaopeng@hrbeu.edu.cn](mailto:gaopeng@hrbeu.edu.cn); and Yujie Xiong; yjxiong@ustc.edu.cn

† These authors contributed equally to this work.

**Supplementary Methods**

X-ray powder diffraction (XRD)

XRD characterization used a Cu-Kα radiation source with a wavelength of λ=0.15405 nm. Voltage and current in the process of testing were 40 kV and 150 mA, respectively. The scanning speed was 10°s^-1^ and the scanning area was from 10° to 70°.

Transmission electron microscopy (TEM)

Transmission electron microscopy (TEM), high-resolution transmission electron microscopy (HRTEM) and scanning transmission electron microscopy with an energy dispersive X-ray (STEM-EDX) spectroscopy characterizations were performed on a FEI Talos F200X G2 transmission electron microscope with a field emission gun operated at 200 kV.

Aberration-corrected transmission electron microscopy (AC-TEM)

JEM-ARM300f was equipped with spherical aberration corrector independently developed JEOL. ETA (expanding trajectory error corrector) was a 12-pole spherical aberration corrector developed by JEOL at an accelerating voltage of 300 kV.

Fourier transform infrared (FT-IR) spectroscopy

FT-IR spectra were recorded on a Perkin-Elmer 580B IR spectrophotometer using the KBr pellet technique.

In-situ FT-IR spectroscopy

In-situ FT-IR spectroscopic measurements for photocatalytic process in the presence of trace water were carried out on the Bruker TENSOR II FTIR spectrometer. Specifically, 30 mg of the photocatalyst was placed on the sample holder in the test chamber, and 100 μL of the H_2_O was injected into the chamber. After the chamber was purged with Ar gas for 30 min, the background spectrum was recorded from 4,000 to 400 cm^-1^. Subsequently, the test chamber was heated to 423 K for 30 min to achieve that the H_2_O molecules were volatilized and adsorbed on the photocatalyst. After the excess vapor of H_2_O was purged by Ar gas, the time-dependent DRIFTS spectra were recorded to monitor the photocatalytic process under the irradiation of Xe lamp and infrared lamp.

Brunauer Emmett Teller (BET) measurement

N_2_ adsorption/desorption measurements were conducted with a Micromeritics TriStar 3020 to characterize the Brunauer Emmett Teller (BET) parameters of samples. The test temperature was 196.15 °C (liquid nitrogen temperature), and the samples were heated at 120 °C for 2 h before the test.

Raman spectroscopy

Raman spectra were taken using a Raman spectrometer (LABRAM HR800) with a 325 nm laser excitation. The samples were dispersed in ethanol for 30 min.

In-situ Raman spectroscopy

In-situ Raman spectroscopic measurements were performed at room temperature using a HORIBA LabRam spectrometer with a 785 nm excitation wavelength at 0.5 mW between 200 and 2500 cm^-1^. To avoid laser-induced deintercalation and photochemistry, the laser power was kept below 0.5 MW and the laser spot size of 1 mm (Olympus LMPlanFl 50*, NA 0.50). A charge-coupled device was used to detect the signal after analyzing the signal via a monochromator. The spectrometer was calibrated in frequency using a HOPG crystal.

X-ray photoelectron spectroscopy (XPS)

XPS was used to characterize the atomic composition content and the chemical valence state, which was equipped with an excitation source of 1486.6 eV of A1Kα target.

In-situ XPS

Samples were analyzed using the Thermo Scientific K-Alpha XPS equipped with MAGCIS (Monatomic and Gas Cluster Ion Source), and the Thermo Scientific ESCALAB 250 Xi XPS parallel images were collected using the ESCALAB 250Xi. The instrument was equipped with a monochromated Al Kα X-ray source, high energy resolution electron analyzer, parallel imaging detector, magnetic immersion lens for improved spatial resolution, and charge neutralization system for insulating sample analysis. The K-Alpha was used for other XPS analyses. Surface layers were removed by bombardment with 4 kV, 2000 atom, Ar gas clusters (8 nA beam current), and scanned over an area of 2 mm 4 mm. Advantage software was used for instrument control & data reduction. The wet catalyst containing water was dropped onto a very transparent silicon wafer. Subsequently, the samples were treated with argon plasma for 10 min and stored in argon atmosphere before further characterization. After the sample was sent to the analysis room, high-purity nitrogen was introduced. A partial pressure of up to 0.5 mbar was introduced into the analysis chamber in turn. A 300 W xenon lamp (PLS-SXE300, perfect light) was used to irradiate the catalyst on the silicon wafer. Ti2p, C1s and O2s spectra were obtained in situ every 5 min.

Photoluminescence (PL) spectroscopy

PL spectra were measured at an excitation wavelength of 320 nm by using a spectrophotometer (FLS 980) and a Xe lamp (450 W) as excitation sources. The time-resolved transient PL decay curve was mathematically described as the following biexponential function:

where τ1 and τ2 are fluorescence lifetimes, and A1 and A2 are corresponding amplitudes. The calculation formula for average fluorescent lifetime is described as follows:

UV-vis reflectance spectroscopy

UV-vis reflectance spectra were collected by an ultraviolet spectrophotometer (UV2550, Shimadzu, Japan).

Electrochemical impedance spectroscopy (EIS)

EIS experiments, photocurrent density characterization and Mott-Schottky analysis were performed using a three-electrode system in a 0.5 M Na_2_SO_4_ solution with Pt foil and saturated Ag/AgCl as the counter electrode and reference electrode by an electrochemical workstation CHI 660D (Chen Hua, China). The calculation formula for carrier density is described as follows:

Surface photovoltage (SPV) measurement

Surface photovoltage measurements were performed at room temperature using a CEL-SPS1000 spectrometer with a 500 W Xe lamp. The catalysts were suspended by a sonicated treatment and dropped on FTO glass.

Photocatalytic water splitting measurements

The photocatalytic experiments were performed in a 500 mL closed quartz flat-bottom container. Firstly, 50 mg catalyst was dispersed with 100 mL distilled water. A 300 W xenon arc lamp was used as a visible light source to trigger the photocatalytic reaction. The power density of the incident light was controlled as 100 mW cm^-2^, which was measured by a CEL-HFX300 Visible spectrophotometer. The amount of gas evolution was measured using an on-line gas chromatograph (SP7800, TCD, molecular sieve 0.5 nm, N_2_ carrier, Beijing Keruida Limited). The solar-to-hydrogen (STH) efficiency (η_STH_) was determined by the following equation:


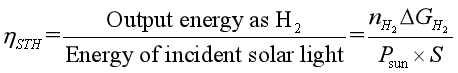


where is the rate of hydrogen production, is the change in Gibbs free energy per mol of H_2_ (at 25°C, G = 237 kJ mol^-1^), P_sun_ is the effective input energy flux of the sunlight (100 mW cm^-2^) and S is the area of the reactor (4.5 cm^2^).

Photocatalytic CO_2_ reduction reaction measurements

The photocatalytic CO_2_ reduction reaction was carried out in a top irradiation vessel connected to the glass gas circulation system of CEL-PAEM-D6 (Beijing China Education Au-Light CO., Ltd). The system was irradiated under an infrared light (>800 nm) with an intensity of 100 mW cm^-2^. Then 50 mg photocatalyst dispersed in 100 ml decolonized water was added in the above system with constant magnetic stirring. The collected gas was analyzed by GC-7920 gas chromatography equipped with thermal conductivity detector and 5A molecular sieve column.

Apparent quantum efficiency (AQE) determination

The apparent quantum efficiency was measured using the photocatalytic reactor setup according to the following equations:

Apparent quantum efficiency (%) = $\frac{Moles of reacted electrons per unit time}{Moles of incident photons per unit time}$ × 100

Mols of incident photons per unit time (NEinstein) = Number of incident photons per unit time/NA. Number of incident photons Np per unit time can be calculated by:

Np = $\frac{Intensity (E)}{Photo energy (Ep)}$

E = Irradiance × reactor area illuminated; and photon energy (Ep) =$\frac{\mathrm{hc}}{\lambda}$ .

Analysis of grain size by XRD

D_hkl_ = Kλ / (B_hkl_ cosθ)

where D_hkl_ is the crystallite size in the direction perpendicular to the lattice planes, hkl is the Miller indice of the plane being analyzed, K is a numerical factor frequently referred to as the crystallite-shape factor, λ is the wavelength of the X-rays, B_hkl_ is the width (full-width at half-maximum) of the X-ray diffraction peak in radians and θ is the Bragg angle.


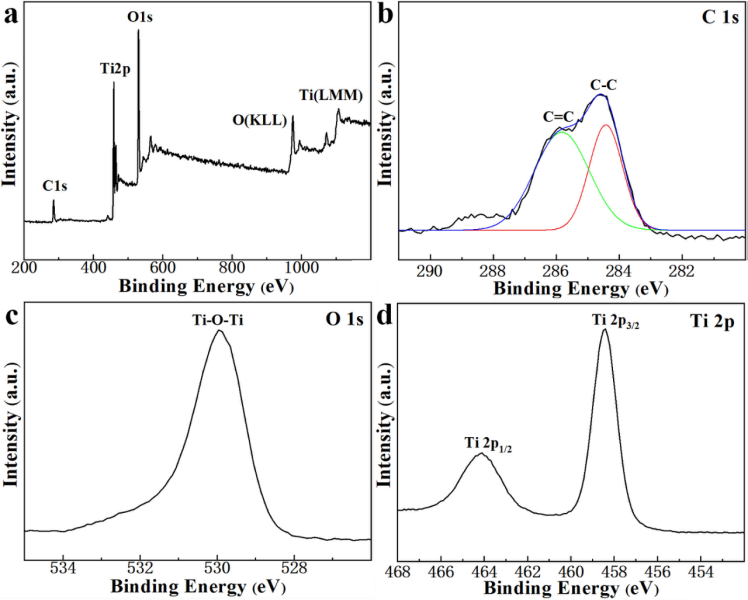


Fig. S1. XPS spectra of the TiO_2_/GQDs: a) survey, b) C 1s, c) O 1s and d) Ti 2p.


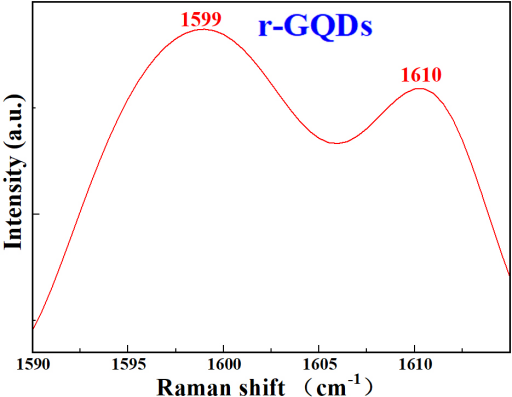


Fig. S2. Raman spectrum of the obtained r-GQDs.


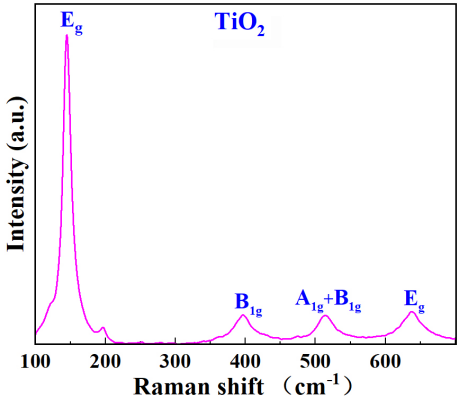


Fig. S3. Raman spectrum of the obtained TiO_2_.


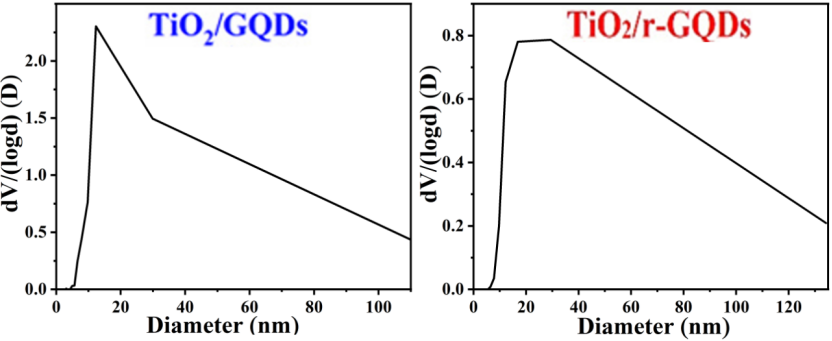


Fig. S4. Pore size distributions of the TiO_2_/GQDs and TiO_2_/r-GQDs.


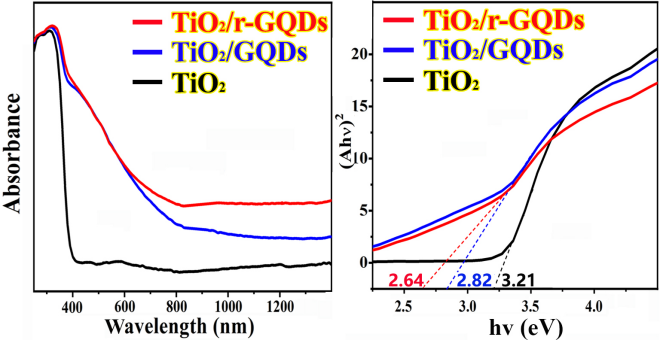


Fig. S5. UV–vis reflectance spectra and the optical band gaps of three samples.


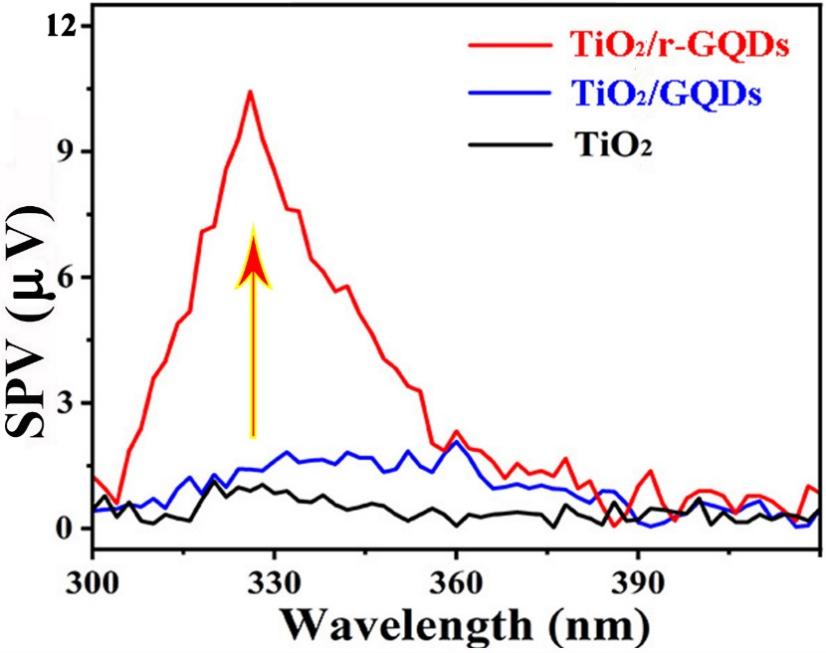


Fig. S6. SPV spectra of three samples.


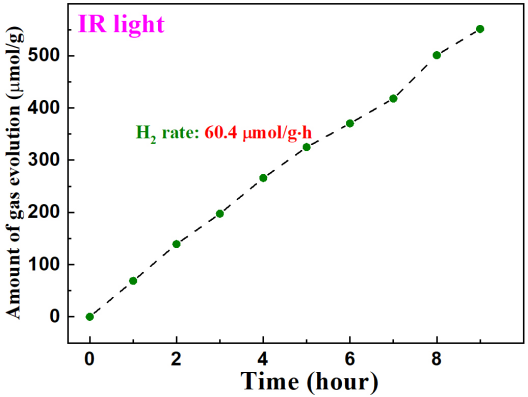


Fig. S7. IR light (>800 nm, 20 mW cm^-2^) driven photocatalytic water splitting for H_2_ generation in 9 hours by the TiO_2_/r-GQDs.


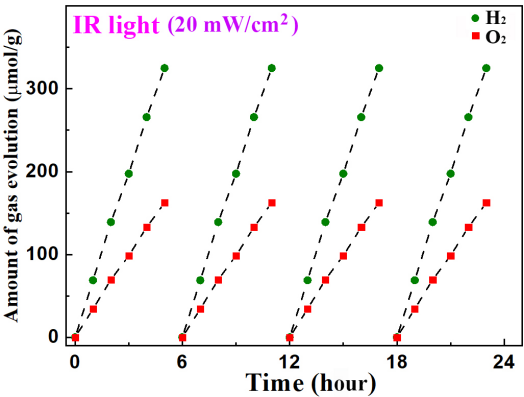


Fig. S8. IR light (>800 nm, 20 mW cm^-2^) driven photocatalytic water splitting for H_2_ and O_2_ generation in 4 repeated cycles (5 h/cycle) by the TiO_2_/r-GQDs.


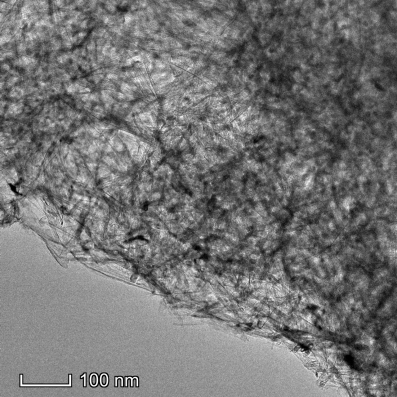


Fig. S9. TEM image of the TiO_2_/r-GQDs after photocatalytic water splitting, which maintains its one-dimensional structure.


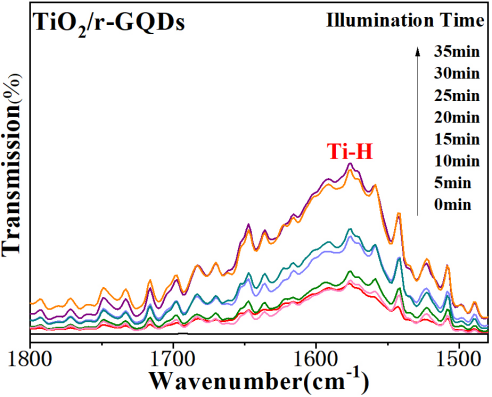


Fig. S10. In-situ FT-IR spectra between 1480 and 1800 cm^-1^ of the TiO_2_/r-GQDs sample, which clearly shows the peaks belonging to Ti-H.


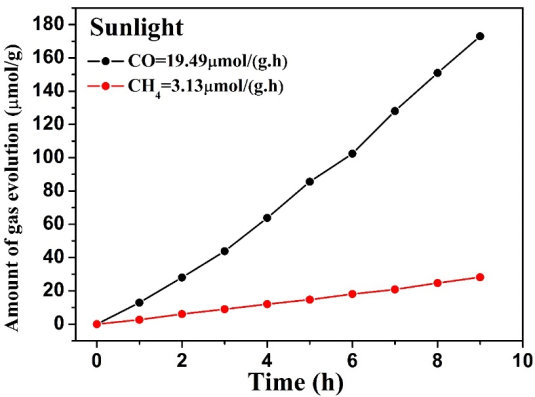


Fig. S11. Simulated sunlight (100 mW cm^-2^) driven photocatalytic CO_2_ reduction for CO and CH_4_ generation in 9 hours by the TiO_2_/r-GQDs.


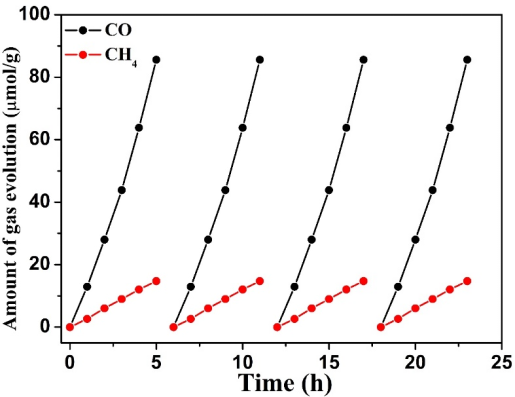


Fig. S12. Simulated sunlight (100 mW cm^-2^) driven photocatalytic CO_2_ reduction for CO and CH_4_ generation in 4 repeated cycles (5 h/cycle) by the TiO_2_/r-GQDs.


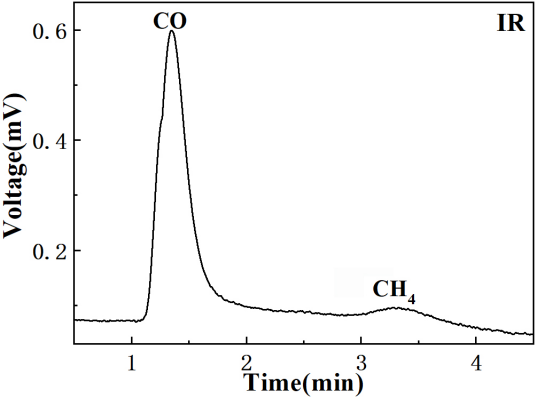


Fig. S13. Gas chromatography measurement for IR light (>800 nm, 20 mW cm^-2^) driven photocatalytic CO_2_ reduction for CO and CH_4_ generation by the TiO_2_/r-GQDs.


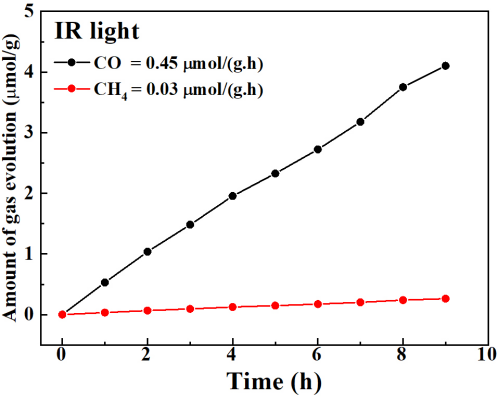


Fig. S14. IR light (>800 nm, 20 mW cm^-2^) driven photocatalytic CO_2_ reduction for CO and CH_4_ generation in 9 hours by the TiO_2_/r-GQDs.


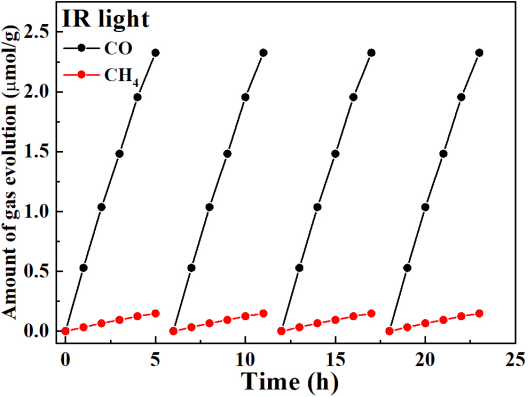


Fig. S15. IR light (>800 nm, 20 mW cm^-2^) driven photocatalytic CO_2_ reduction for CO and CH_4_ generation in 4 repeated cycles (5 h/cycle) by the TiO_2_/r-GQDs.
